# Supplementary material for: Identification of Flavone Derivative Displaying a 4′-Aminophenoxy Moiety as Potential Selective Anticancer Agent in NSCLC Tumor Cells
Source: Molecules. 2023 Apr 5;28(7):3239. doi: 10.3390/molecules28073239 (PMC10096842; doi:10.3390/molecules28073239)

**Supporting Information**

# Flavone derivative displaying a 4′-aminophenoxy moiety as potential selective anticancer agent in NSCLC tumour cells

**Giovanna Mobbili[1], Brenda Romaldi[2], Giulia Sabbatini[1], Adolfo Amici[2], Massimo Marcaccio[3], Roberta Galeazzi[1], Emiliano Laudadio[4], Tatiana Armeni[2], Cristina Minnelli[1*]**

[1] Department of Life and Environmental Sciences, Marche Polytechnic University, 60131 Ancona, Italy; g.mobbili@univpm.it, giulia.sabbatini@staff.univpm.it, r.galeazzi@univpm.it, c.minnelli@staff.univpm.it
[2] Department of Specialist Clinical Sciences, School of Medicine, Marche Polytechnic University, 60131 Ancona, Italy; b.romaldi@pm.univpm.it, t.armeni@univpm.it, a.amici@staff.univpm
[3] Department of Chemistry G. Ciamician, University of Bologna, Via Selmi 2, Bologna 40126, Italy; massimo.marcaccio@unibo.it
[4] Department of Science and Engineering of Matter, Environment and Urban Planning, Marche Polytechnic University, 60131 Ancona, Italy; e.laudadio@univpm.it

* Correspondence: c.minnelli@staff.univpm.it

**Table of Contents**

**Figure S1. Exposures of western blot analysis……………………………… 2**
**Figure S2. IC50 curves……………………………………………………….. 3**
**Figure S3. Copies of $^{1}H$ NMR and $^{13}C$ NMR Spectra……………………… 4-10**
**Figure S4. Copies of HRMS Spectra……………………………………….. 11-12**

**Figure S1. Exposure of western blot analysis**

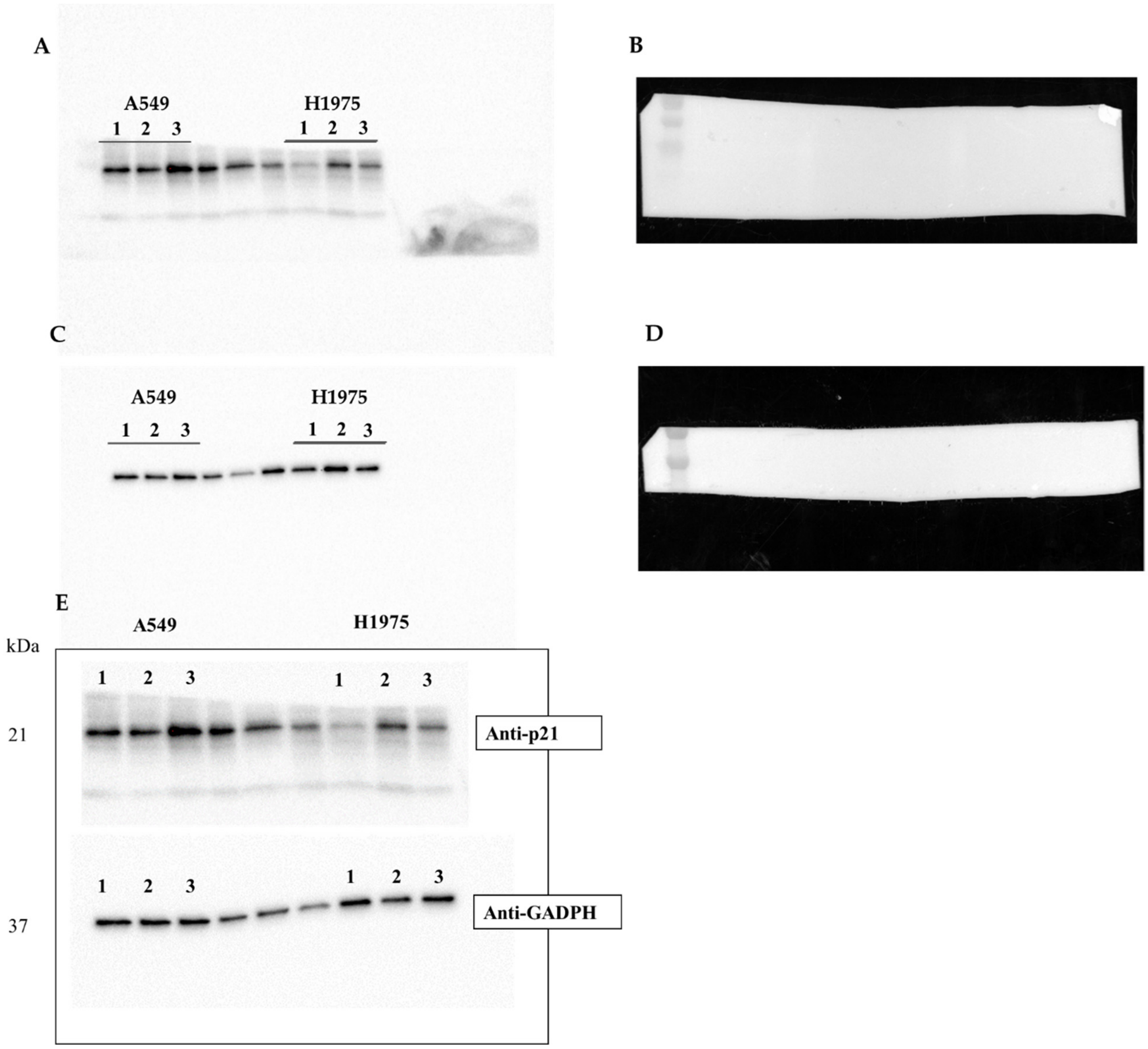


**Exposure of western blot analysis.** The sample derive from the same experiment; gels and blots were processed in parallel. After blot, membranes were then cropped, with the help of the marker, at the height of the protein of interest before being incubated with the antibody. During development at Chemidoc, the cropped membranes were developed together considering the sensitivity of the antibody and the same exposure times. Cropped membrane for p21 (a) and GADPH (c); Membrane marker for p21 (b) and GADPG (d). Samples, **1**-untreated; **2**-APF-1, 1.5 μM; **3**-APF-1, 3 μM.

**Figures S2 and S3. Copies of $^{1}$H NMR and $^{13}$C NMR Spectra**

NMR spectra of compound **APF-2** (**$^{1}$H NMR**, 400 MHz, DMSO-d6)

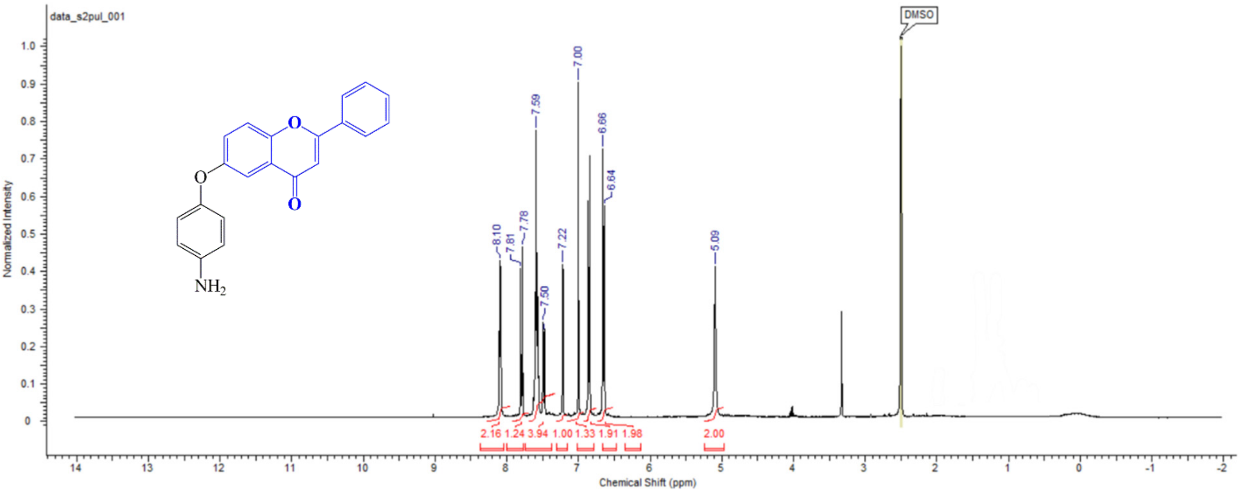


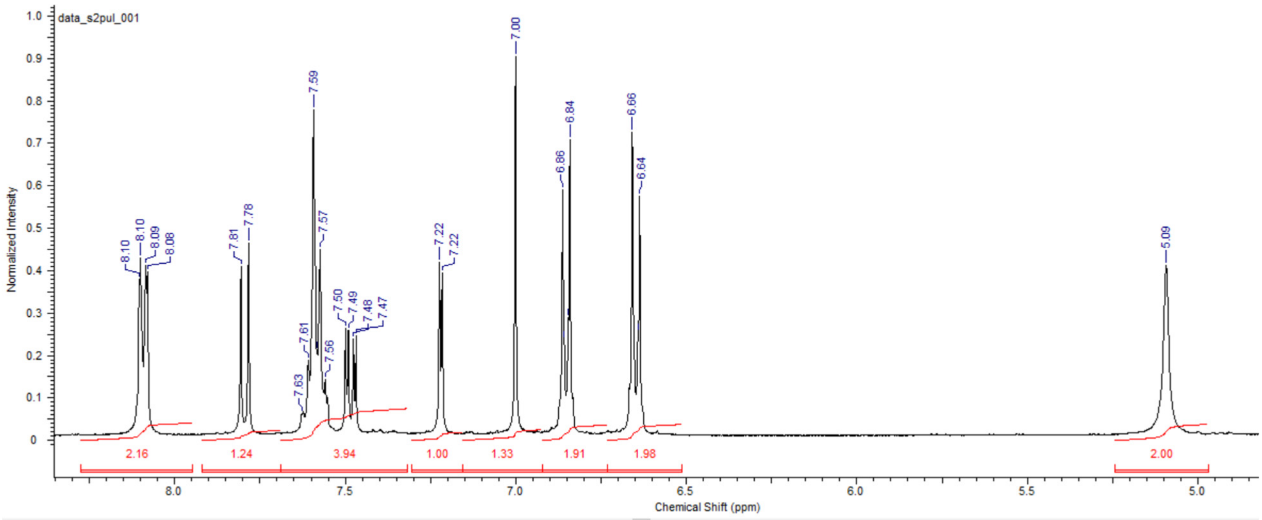

NMR spectra of compound **APF-2** ([13]**C NMR**, 100 MHz, DMSO-d6)

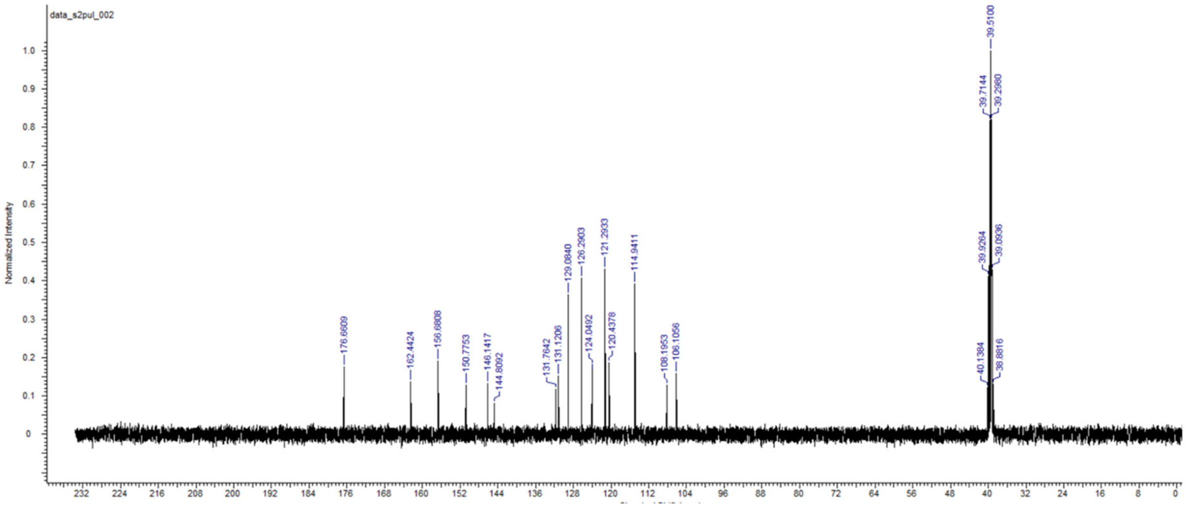


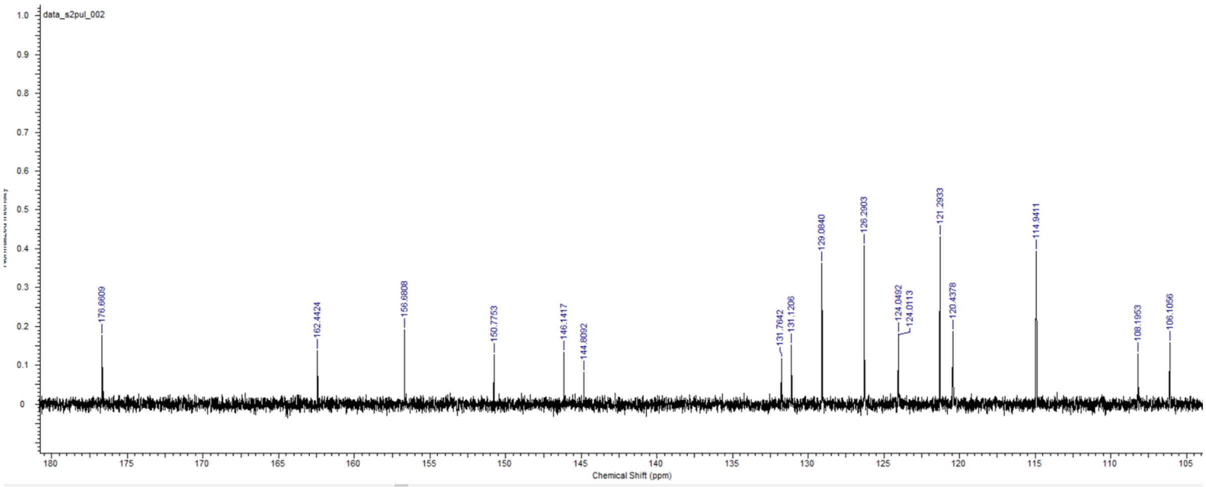


NMR spectra of compound **APF-3** ([1]**H NMR**, 500 MHz, DMSO-d6)

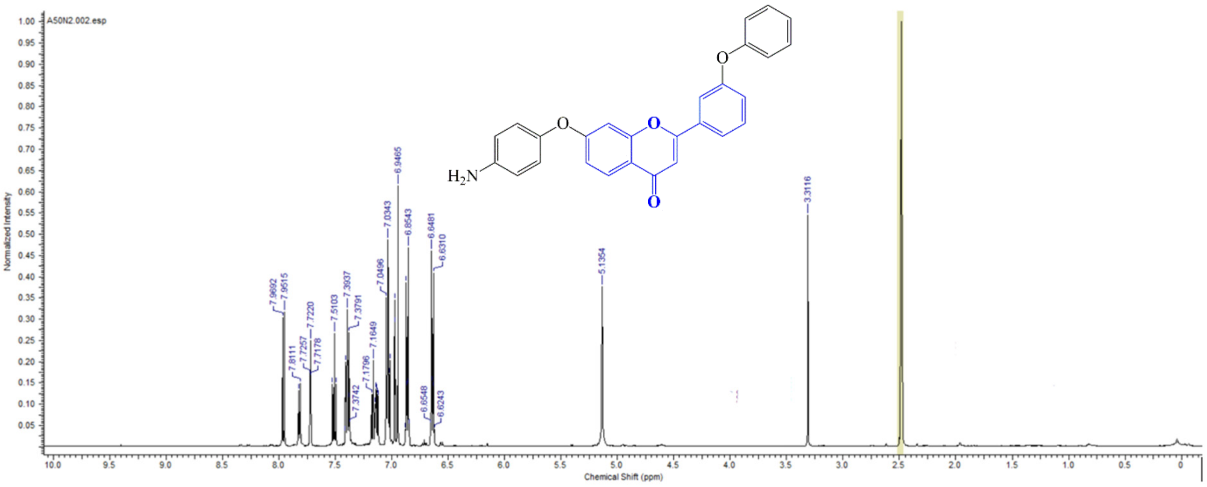

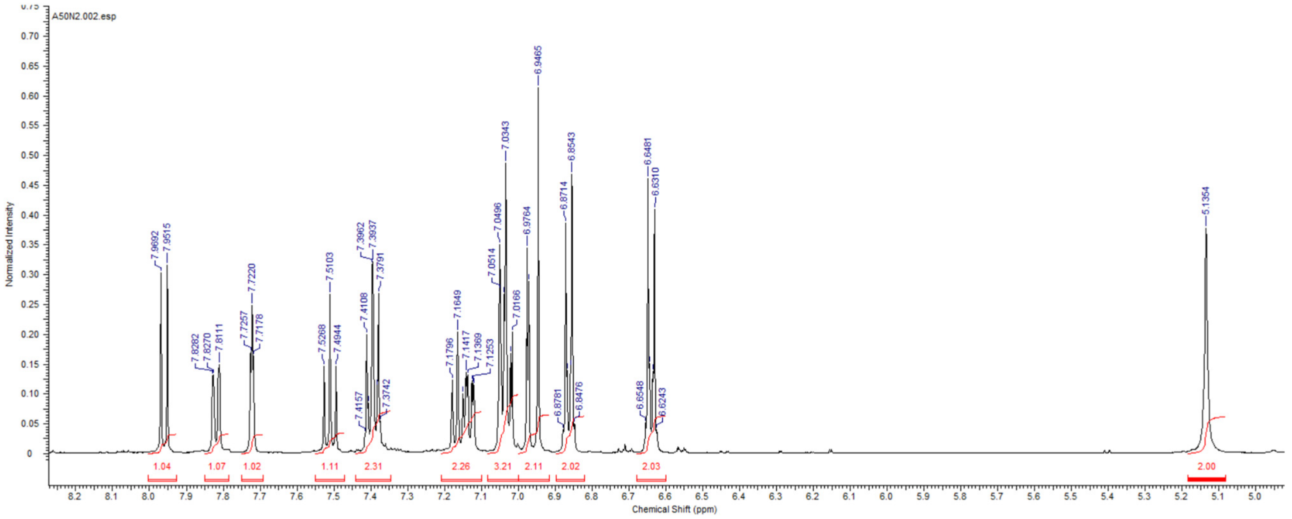


NMR spectra of compound **APF-3** ($^{13}$**C NMR**, 125 MHz, DMSO-d6)

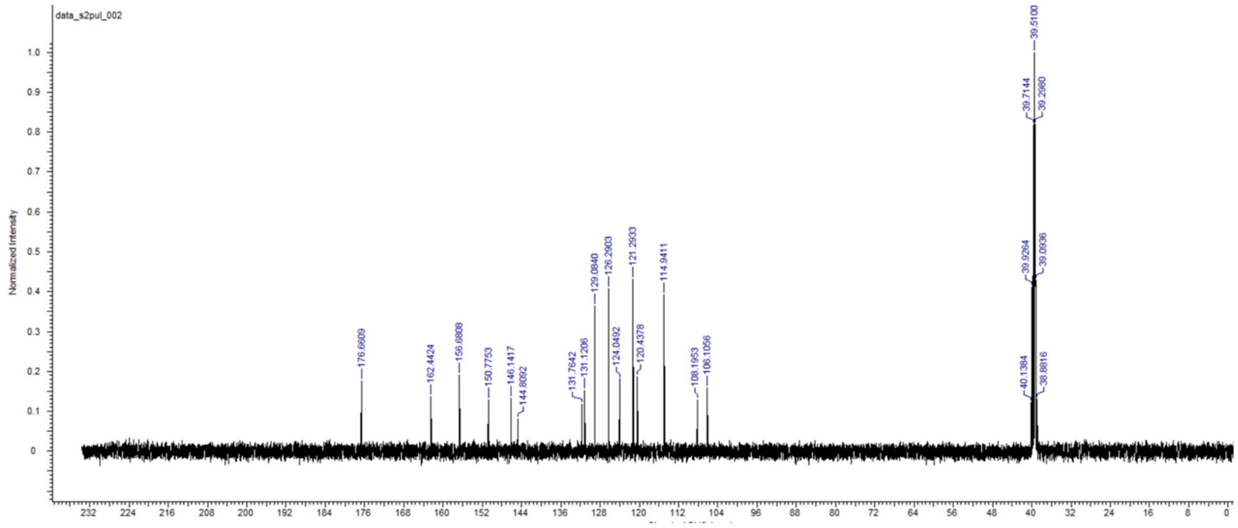


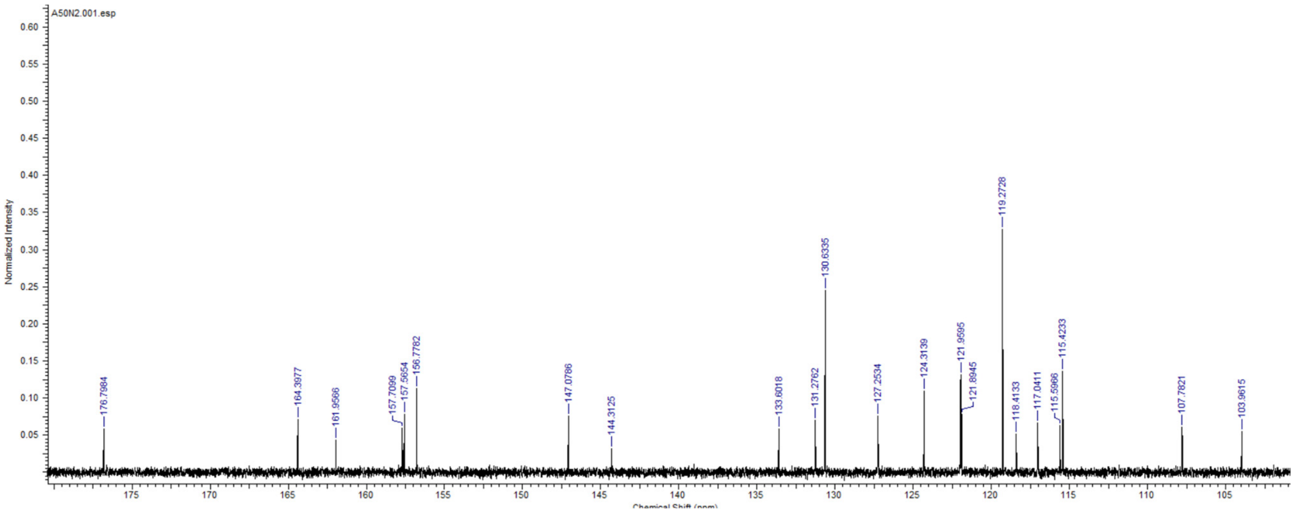

NMR spectra of compound **APF-4** ($^1$**H NMR**, 400 MHz, DMSO-d6)

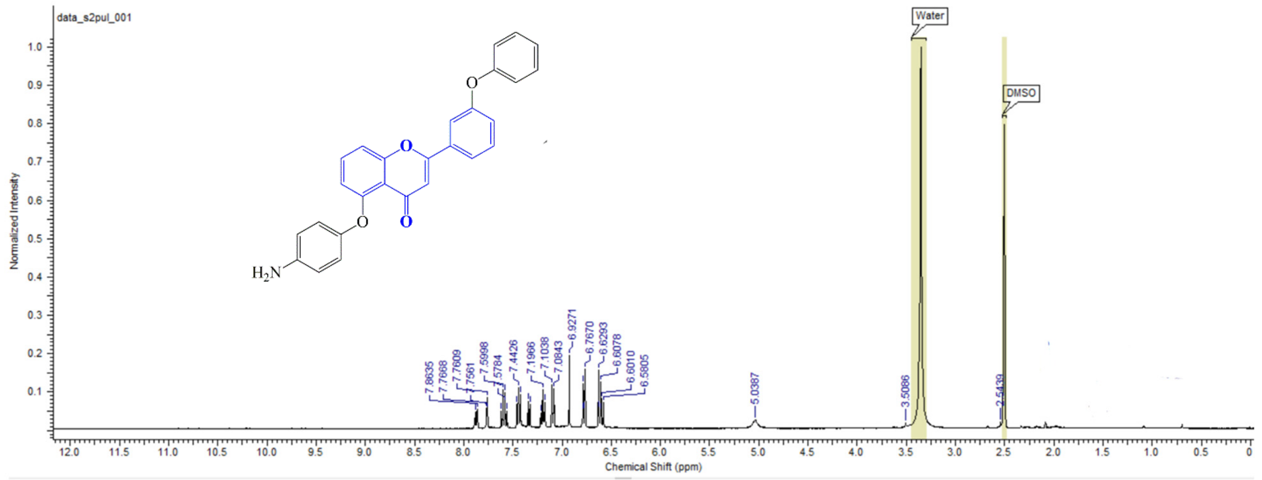


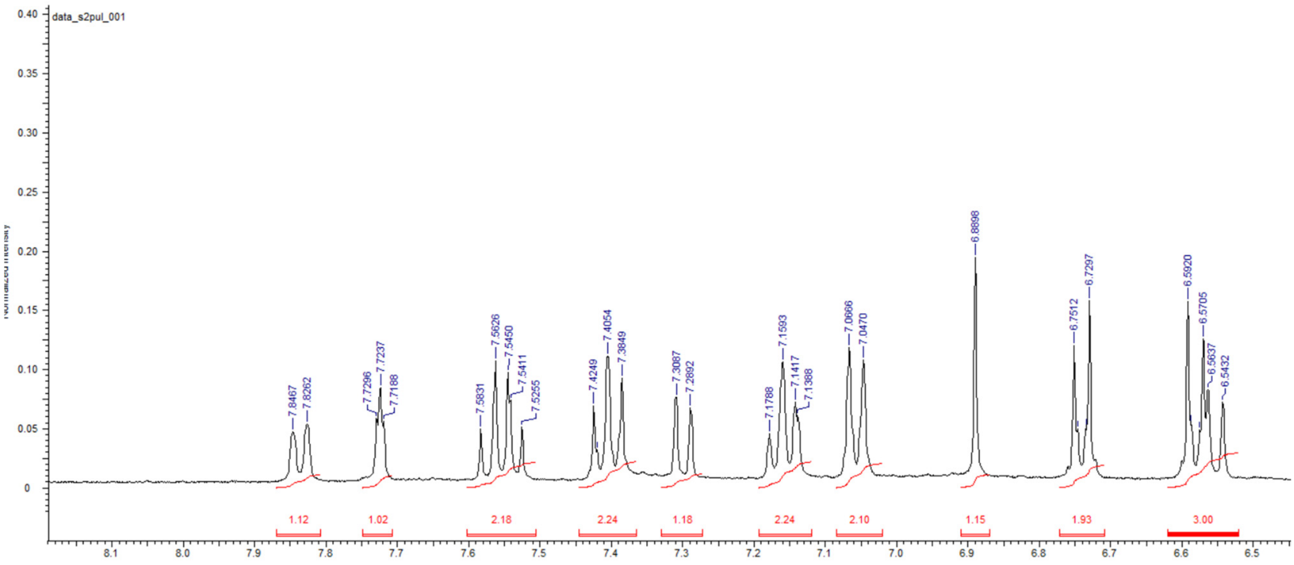

NMR spectra of compound **APF-4** ($^{13}$**C NMR**, 100 MHz, DMSO-d6)

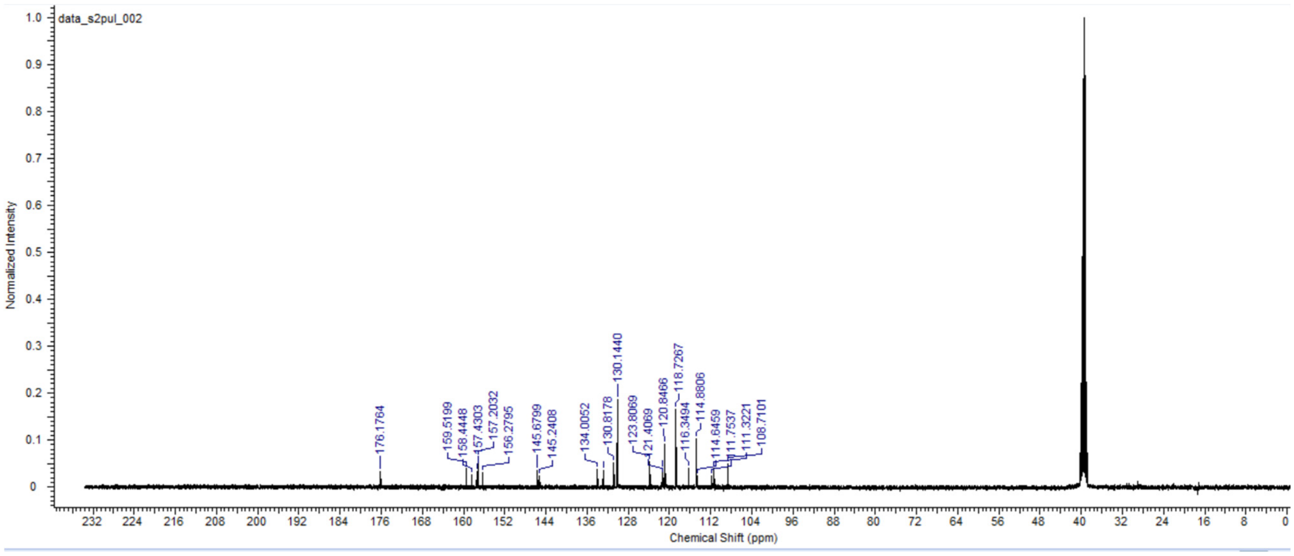


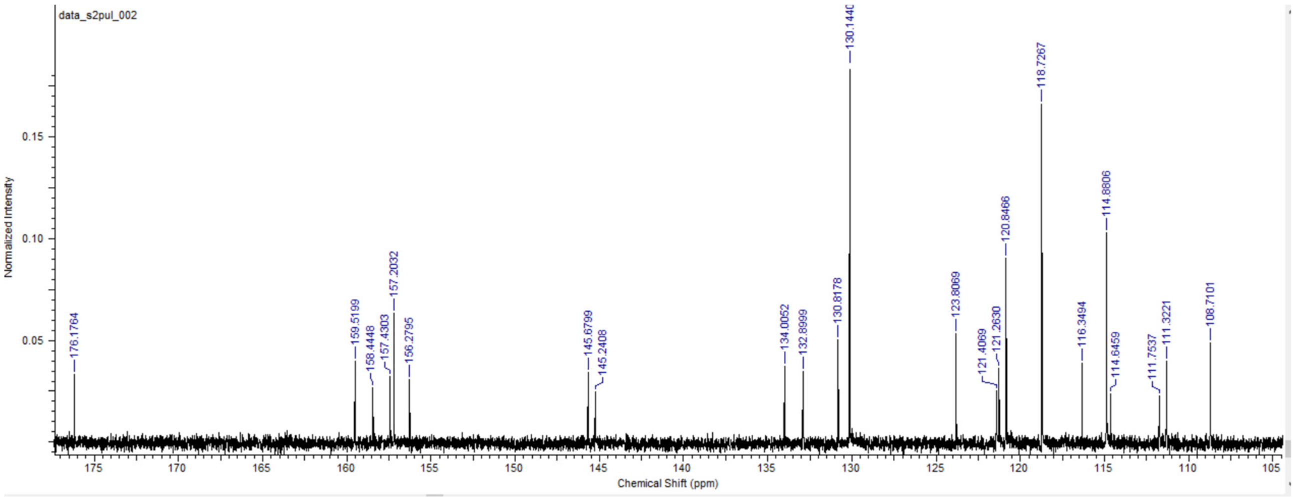

NMR spectra of compound **APF-5** ([1]**H NMR**, 500 MHz, DMSO-d6)

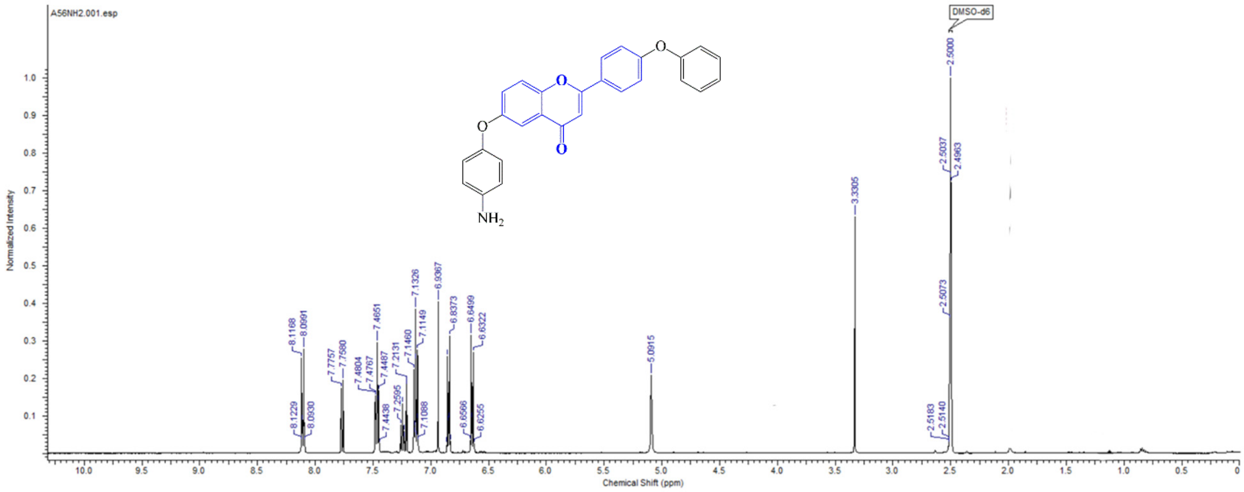


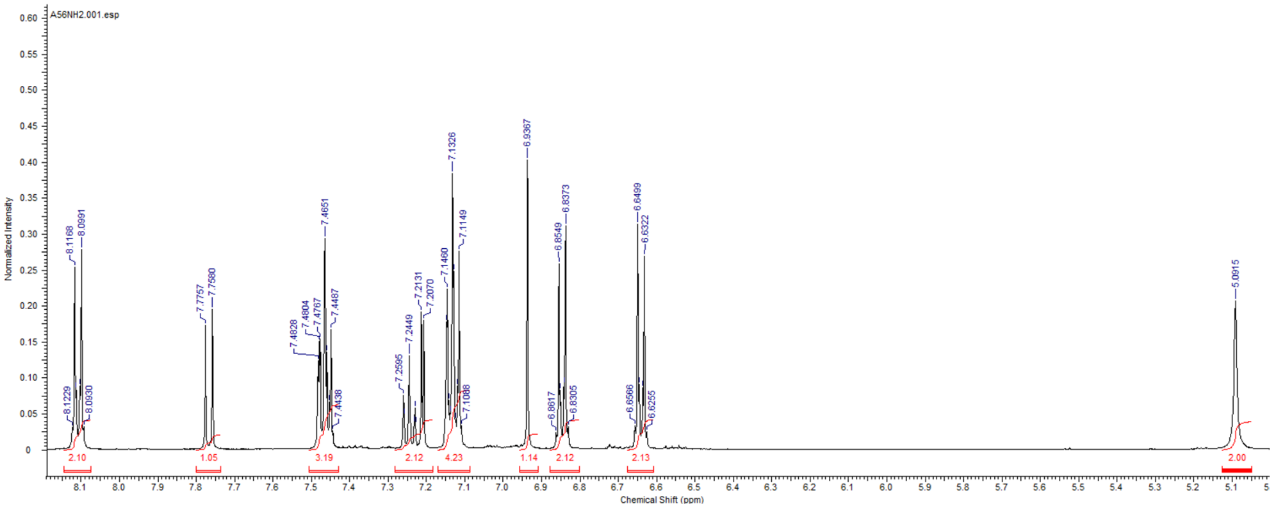

NMR spectra of compound **APF-5** ($^{13}$**C NMR**, 125 MHz, DMSO-d6)

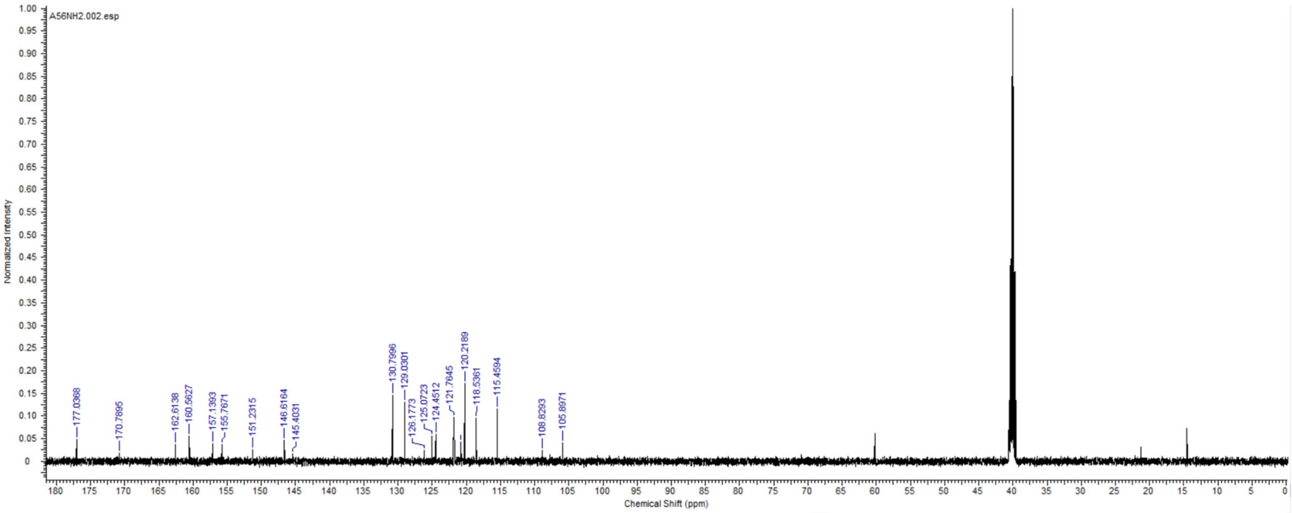


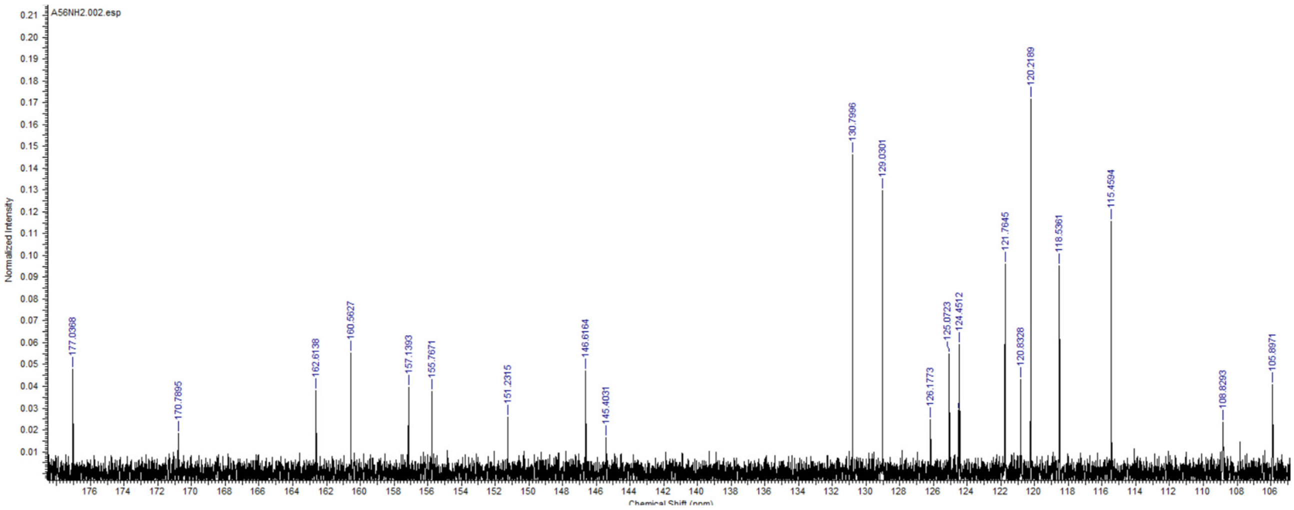

**Figure S4. Copies of HRMS Spectra**

HRMS Spectrum of compound **APF-2** (neutral mass 329. 34723 Da).

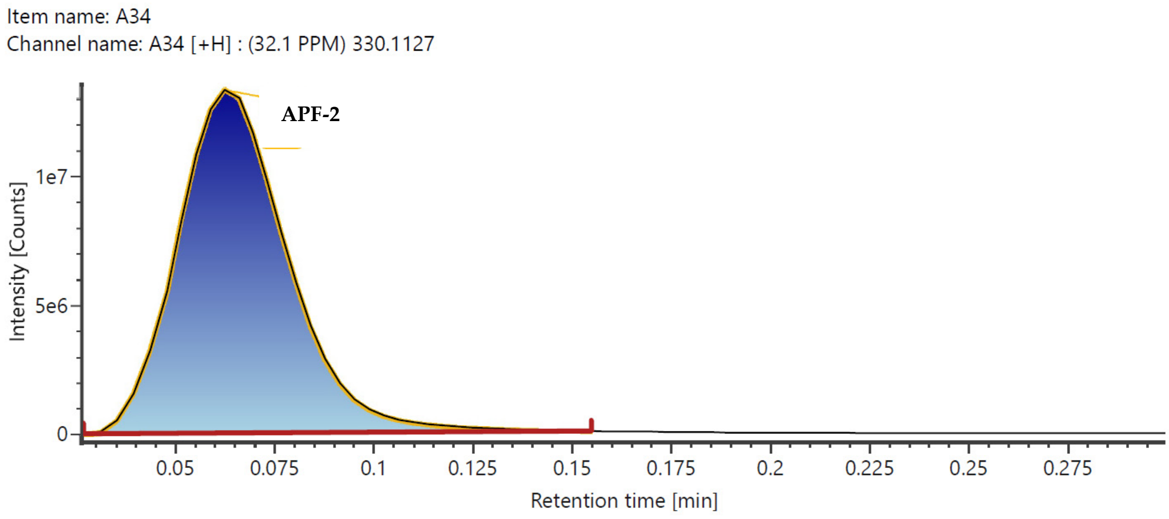


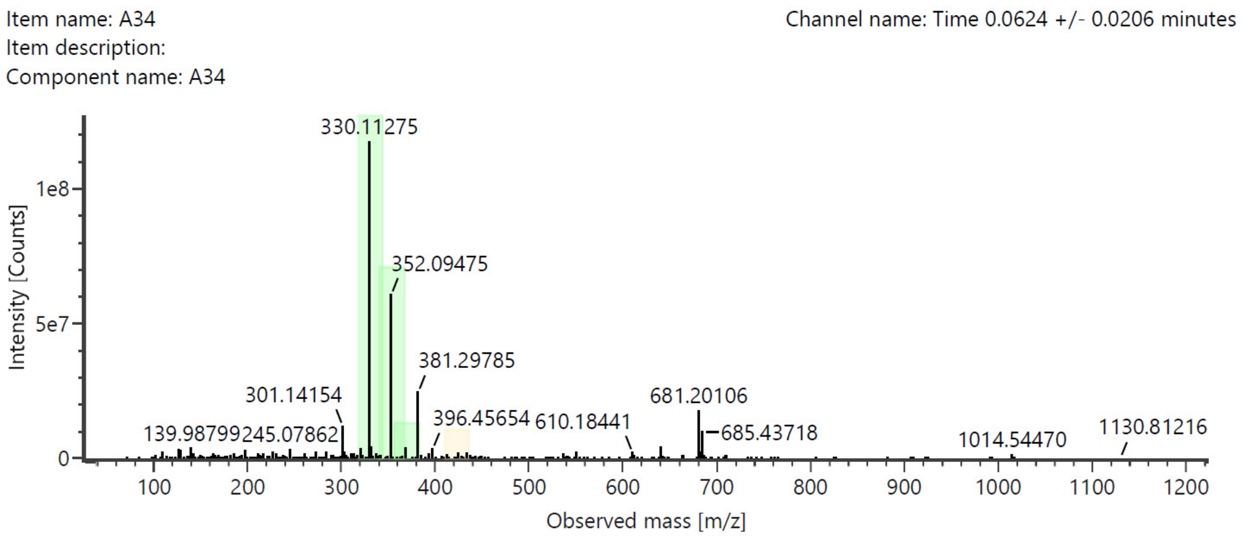


HRMS Spectrum of compound **APF-3** (neutral mass 421.13141 Da)

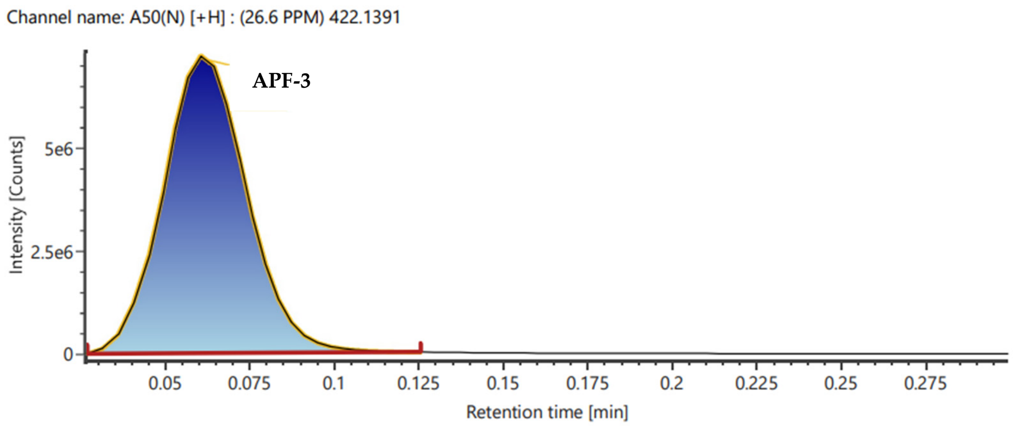

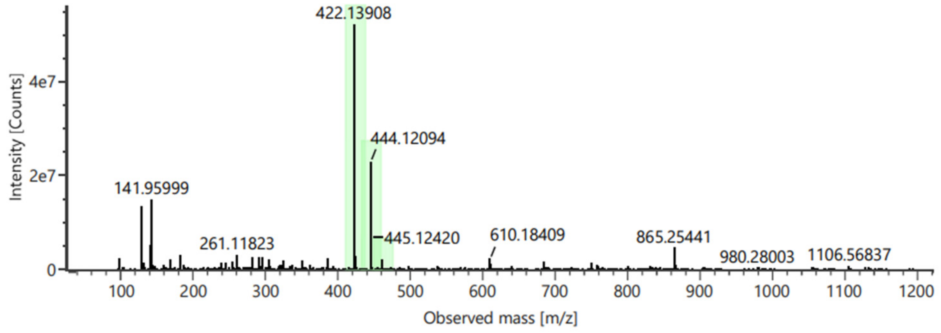


HRMS Spectrum of compound **APF-4** (neutral mass 421.13141 Da)

Item name: A52
Channel name: A52 [+Na] : (26.6 PPM) 444.1205

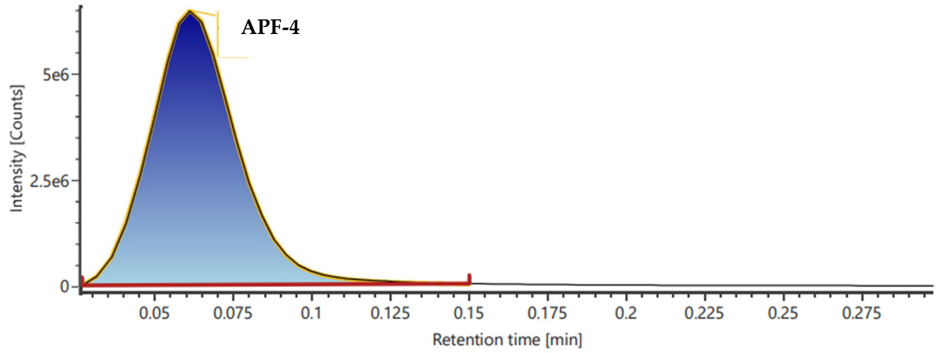


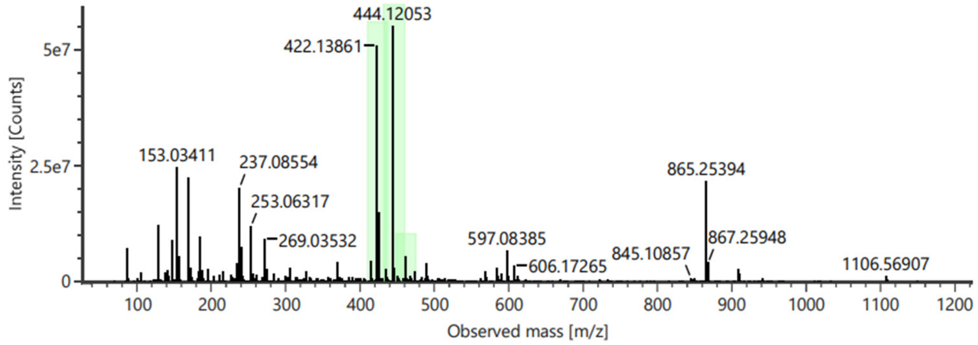


HRMS Spectrum of compound **APF-5** (neutral mass (neutral mass 421.13141 Da)

Item name: A56-NH2
Channel name: A56-NH2 [+H] : (26.6 PPM) 422.1389

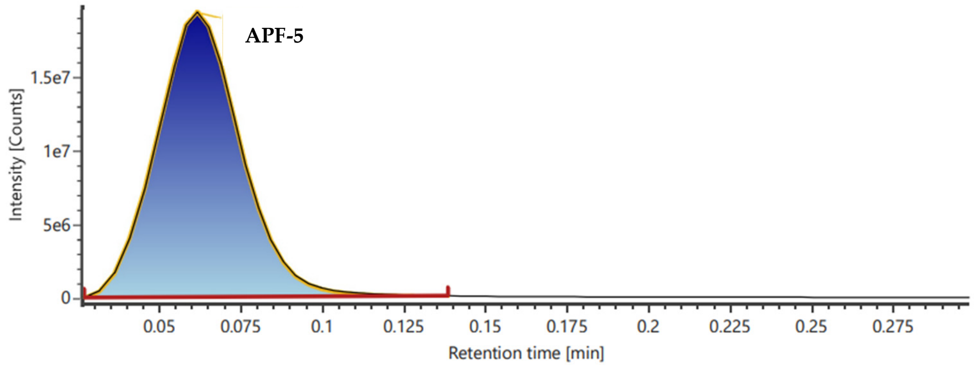

Item name: A56-NH2
Item description:
Component name: A56-NH2

Channel name: Time 0.0615 +/- 0.0209 minutes

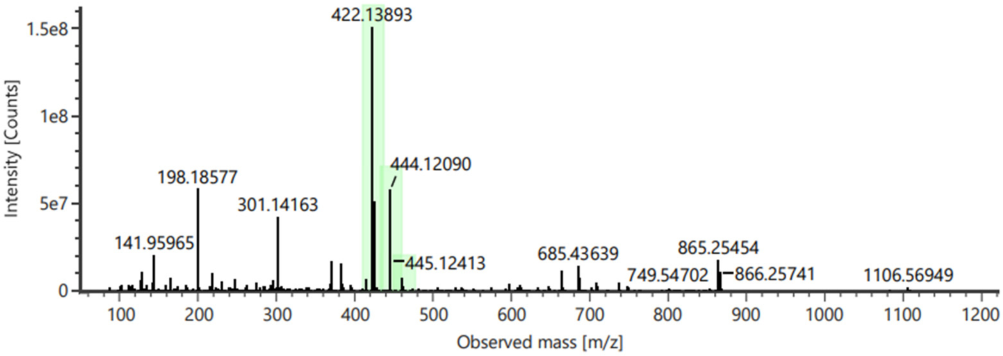

Supplement: Supplementary file 1 [file molecules-28-03239-s001.zip › molecules-2306010-supplementary-latest.pdf]
